# Life Engineering: Towards a new discipline

**DOI:** 10.1007/s12599-020-00680-x

**Published:** 2021-01-05

**Authors:** Rainer Alt, Andreas Göldi, Hubert Österle, Edy Portmann, Sarah Spiekermann

**Affiliations:** 1 Leipzig University, Leipzig, Germany; 2 Btov Partners, St. Gallen, Switzerland; 3 University of St. Gallen, St.Gallen, Switzerland; 4 University of Fribourg, Fribourg, Switzerland; 5 Vienna University of Economics and Business, Vienna, Austria

## Introduction

Rainer Alt

The last decade has brought about a major change in the diffusion and the role of information technologies (IT). First, they have moved from backstage to frontstage. They are no longer “only” supportive for a firm’s products and processes, but they are now core elements that determine the success of almost any firm in the marketplace (Matt et al. 2015). Second, they have become ubiquitous. IT is no longer limited to the firm, but present in the life of individuals and in the entire society. In fact, there is a reversal in the relationship between individuals and computing resources. The power large computers had some ten years ago is now present at the wrist of individuals and physical objects such as cars and homes have turned into computer systems. While in the early days of computing multiple users accessed one device (i.e., a mainframe or workstation computer), now every single user accesses multiple devices. Following a recent report of the consulting company Deloitte, US households have an average of 11 connected devices (Westcott et al. 2019). These devices are more closely engrained in the daily lives of their users than ever before and have permeated their daily routines. Among the examples are the consumption of medial content, the planning and support of mobility, the monitoring of personal activity, or simply the execution of commercial transactions and social interactions online.

### Two Sides of the Digital Economy

Following from the observation that IT in general and artificial intelligence in particular are general purpose (Brynjolfsson and McAfee 2017) as well as dual use (Evan and Hays 2006) technologies, the implications are ambiguous. Depending on the perspective and the interpretation, they may point in a positive or a negative direction. Notwithstanding all attributions and clearly to observe is the economic power: The digital economy has become a dominant factor in the economic world having created wealth and employment alike. Today, the largest companies are digital businesses, and the combined value of digital platform businesses with a market capitalization of > 100 million USD alone has grown by 67% between 2015 and 2017 to more than seven trillion USD (UNCTAD 2019). This development has given birth to many valuable services that empower users in their various roles. Consumers now have access to the same tools (e.g., catalogs, ratings, stock quotes) as the professional staff of the service providers, a trend to be observed in many industries, from news, retailing and finance to medicine. In the latter, numerous services have emerged that contribute to well-being and patients’ independence. Even the laggard public sector has embarked on digitally interacting with and thus empowering citizens.

At the same time, albeit the services might be free of charge, they come at a price. Providers of such digital services have unprecedented access to user data, which on the one hand creates the value of data-based (smart) services, but leads to concerns regarding privacy or manipulation on the other. A well-known phenomenon is the privacy paradox, which denotes a variety of (often non-rational) trade-offs (Gimpel et al. 2018). The large big tech businesses, which operate digital platforms that accumulate data from services and devices to continuously improve their offerings are in a pole position with regards to interacting with individuals. Although they are already present in many areas of our daily life, it might be a legitimate assumption that this happens not primarily in the interest of their users, but in the vested interest to maximize their own market position as well as their revenues (e.g., van der Aalst et al. 2019). Worrying are not only the biases in the market (e.g., barring competitors from platform access) and in the offerings (e.g., luring or nudging of users to use services, spreading of false information), but also the use for repression by governments (e.g., social scoring or censorship) or a variety of alarming social impacts. Among the latter are excessive social media, gaming or smartphone use, which have been recognized as sources of depression (e.g., Baker and Algorta 2016; Cudo et al. 2020; Kuss and Griffiths 2017), or the negative effects of information overload and technostress on life quality and happiness (e.g., Binswanger 2006).

### Directions to Life Engineering

Understanding and designing IT in the business world has been at the heart of the business and information systems engineering (BISE) domain for many decades with many frameworks and methodologies that have also been adopted in practice (e.g., Österle 1995; Scheer 1998). In view of the changing role and presence of IT, “fundamental change in the BISE landscape” was deemed necessary, involving a shift from the dominant business perspective to the perspective of the digital user “as a new field of BISE” (Brenner et al. 2014, p. 55f). The interdisciplinary tradition and the differentiated methodologies of the BISE discipline are assets in this direction. For example, multiple perspectives are valuable when addressing the growing complexities of the socio-technical design task. They reflect the conviction that technological infrastructures like ERP systems or mobile devices will only create value when aligned with value-adding applications and services. In addition, the name of the BISE discipline carries the term “engineering”, which implies to systematically approach the design of these systems. Along these lines “Life Engineering” may be seen as a successor of the “Business Engineering” approaches, which already recognized the role of customer processes in the context of enterprise systems. Life Engineering has been coined to explicitly address the design of IT from the user’s perspective and in the user’s interest. It builds on digital solutions that have been developed to support users in “living their life”, but must not be confused with approaches from the natural sciences that aim at engineering human life (e.g., Baker et al. 2006).

A concept that is applied in many user-facing digital solutions today is that of (personal) digital assistants. By supporting the interaction between a user and one or more service providers, many of such assistants are expected to emerge in various areas of life (e.g., commerce, education, health, mobility) featuring a more or less comprehensive scope of service integration (e.g., Alt et al. 2019). However, the question arises how the downsides of the digital economy mentioned above may be prevented. First of all, user-centric solutions for citizens, customers or patients will need to convey the values of these groups, which will most likely differ from those of commercial organizations like the big tech businesses. Thus, a Life Engineering discipline as a separate branch of knowledge will have to understand the values that guide the actors involved in the development of solutions and offer general as well as operational guidance in this process. In addition to the existing concepts from the BISE discipline, several models may provide valuable input. User values may be derived from economics and management (e.g., homo economicus, homo digitalis, see Backhaus and Awan (2019)), from sociology (e.g., social engineering, see Suyanto et al. (2020)), or from medicine (e.g., quality of life, see Bakas et al. (2012)). Many general guidelines and codes of conduct also exist that embody ethical values (e.g., ACM^2018^; IEEE 2019) and led to the notion of engineering ethics (see Nguyen 2020).

Most of these approaches are recommendations on a rather general level. They may be applied by service providers from the private sectors, by government and regulating bodies as well as by user communities. First, businesses could complement existing strategies on corporate social responsibility (e.g., Porter and Kramer 2006) when devising new user-centric services. Among the examples are identity management providers in the private (e.g., bankID, Verimi) and the public (e.g., SwissID, DigiD) sector. Second, initiatives by public authorities could aim at enforcing individual data rights and ethical values like autonomy and sovereignty via regulation. Examples are the recommendations of Germany’s Data Ethics Commission (DEC 2018) or the “Ethics guidelines for trustworthy AI” (EC 2019) as well as the Berlin declaration on digital society (EC 2020a) and the Digital Services Act (EC 2020b) by the European Commission. Third, users might engage themselves in developer communities that push  decentralized solutions (e.g., self-sovereign identies such as Jolocom, Sovrin) and tools to increase personal autonomy and agency referred to as personal information management systems (PIMS, e.g., Abiteboul et al. 2015). Generally, these streams are advances towards incorporating ethical and human values in machine intelligence and towards stronger user sovereignty, but today’s solutions in these directions still remain piecemeal and little aligned.

### Contributions in Discussion Section

To discuss the directions towards a Life Engineering discipline, a panel at the International Conference on Wirtschaftsinformatik (WI2020) collected statements from three scholars and one practitioner. While these esteemed colleagues agreed that the current situation was at a critical crossroads and that a discipline Life Engineering was necessary, they emphasized different aspects.

The first position is by Andreas Göldi, who founded his first IT business in 1996 and is now a partner with the European venture capital firm btov. He asserts that the dominance of big tech companies has negative impacts on society and foresees substantial market opportunities for digital innovations that focus on improving the quality of life. For the “vision of an all-encompassing digital life assistant” he identifies several shortcomings, which need to be addressed in a collaborative effort among businesses, governments and regulators. In his view, holistic frameworks are important enablers in this process.

The second contribution adopts the design-oriented engineering perspective. Hubert Österle from the University of St.Gallen argues that Life Engineering requires similar artefacts to Business Engineering, but points out that the transformation of human lives will be substantially more complex. To tackle this endeavor, a Life Engineering discipline should join forces with other disciplines and strive to make ethical guidelines more operational to be applicable in service development processes. At the same time, he calls to mind that ethical guidelines must not stifle competition and innovation.

A third statement is contributed by Edy Portman from the University of Fribourg. Being a researcher in the field of human-centered interaction science and technology, he sees ethical issues embedded in the interfaces and the business models of digital services. Besides the collaboration of multiple disciplines, he advocates the application of soft computing methods and systems theory to capture and model the factors that influence human life quality. Contrary to the current scenario, users should be able to control their data, which requires new forms of service mandates and trusted intermediaries.

In the fourth and final position, Sarah Spiekermann from the University of Vienna assumes that technology needs to be deliberately pushed in “the right direction”. She introduces the concept of Value-based Engineering, which combines a philosophical and an engineering perspective. The suggested development paradigm devises a discourse among stakeholders in which individual systems of interest emerge. Besides this procedure model, other elements such as the value vocabulary and the value register as well as risk assessment methodologies reflect key elements of the BISE discipline.

In summary, the contributors of this discussion section propose diverse views on how technology may be leveraged for sustaining positive human values and long-term life quality. All four see the need for a collaborative effort among many disciplines and sectors. They also illustrate the dimension of the challenge “engineering life” since “designing” human life will be substantially more intricate than designing business strategies, processes or systems. Life is by nature a complex, vague as well as dynamic construct, and determining whether something is desirable remains often in the eye of the beholder. In any case, treasured values such as individual freedom should be preserved whenever possible and instead of leaving future developments to chance or to the competitive forces of the market, the BISE discipline should take on this task. The contributions show that the discipline is in a good position and it may be expected that existing knowledge on frameworks and architectures, methods and modeling, algorithms and machine learning will prove helpful in advancing the emerging discipline of Life Engineering in a near future.

## Life Engineering: The Commercial Impact and the Need for a Comprehensive Framework

Andreas Göldi

In recent years, the impact of digital technology on our societies and the well-being of humans has increasingly been subject to more scrutiny. Particularly the dominant role of “big tech”, the big five technology companies – Google, Facebook, Amazon, Microsoft and Apple –, is frequently criticized (Galloway 2017). Their influence on modern consumer and business technology sometimes feels almost unlimited, and the resulting questions around privacy, political polarization, mental health and inequality are complex and of essential importance for healthy societies.

However, the debates around these topics are often conducted in an isolated and siloed way, without an understanding for the interconnectedness of these aspects and the role of technology as a broader force with long-term impact that in many ways has a positive impact on the quality of life. The dominant sentiment seems to be one of fear, mistrust and pessimism. This could be interpreted as a reaction to the originally strongly overoptimistic stance toward digital technology (Göldi 2020). Most of all, the question of how digital services can be created that are useful for society and at the same time commercially viable is crucial if we hope that innovation in these fields will develop further.

The concept of Life Engineering as proposed by Hubert Österle (2020c) brings a new and holistic perspective to the table, and it is one that is urgently needed: It first seeks to understand the interconnectedness of human needs on several levels, which is crucial for an analysis of the hoped-for benefits, but it also attempts to detect unexpected consequences of digital technologies.

For example, a seemingly innocent social photo-sharing application such as Instagram seems to primarily appeal to users’ social needs (“community” in Österle’s framework), but has direct implications on people’s emotions around status, self-esteem and even safety, with indirect effects for their health. The resulting challenges for mental health, particularly for younger users, are well documented (Berryman et al. 2018). This in itself is hardly surprising. It is well known from history that technology can have unintended negative consequences. In turn, there are often technological solutions for these negative effects, and the more difficult aspects of our highly digitized modern world are no exception. For example, in recent years many startups in the field of mental health have been founded (Gillet 2020) that try to mitigate (digital) stress – anything from screen-time limiters to meditation apps to encourage behavioral therapy towards general emotional well-being. Many of these apps are highly popular and regularly appear in the top charts of app stores (e.g., Google 2020) – practical evidence for the considerable commercial potential of such approaches that seek to improve quality of life, as limited as they might be for now.

“Big tech” has discovered this need as well. To name just a few examples: Apple and Google have introduced screen-time feedback mechanisms into their latest mobile operating systems to – ironically – encourage people to use their devices less. Microsoft is sending reminders to users of its Office 365 productivity suite to reserve quiet time in their busy schedules. Amazon is actively encouraging healthcare companies to build “skills” for its Alexa voice assistant – an add-on functionality that supports health-related processes.

Are these relatively simple approaches first steps towards more holistic solutions for true Life Engineering? Most of what is on the market today makes the impression of being very limited and isolated, far away from Österle’s vision of an all-encompassing digital life assistant. Even if we just focus on the limited scope of digital health applications (a subset of Österle’s broader picture), there are three important reasons for this somewhat disappointing state of the art.

First, applying technology to the complexity of daily life and health is simply very hard. Apps that try to provide meaningful advice have to deal with imperfect real-world user data from limited sensors such as smartwatches. Many also rely on self-reported data from users, which introduces bias and inconsistency. Furthermore, applying even the most sophisticated machine learning (ML) algorithms to these kinds of datasets is difficult, since most ML approaches are not designed to deal with the discontinuity, complexity and weak signals of real-world events (Ghassemi et al. 2018).

Second, many of these approaches only become meaningful when data and results from different systems are integrated. For example, a holistic picture of an individual’s health and fitness might require data from their smartphone, scales, blood pressure measuring device and sleep monitor, as well as nutritional information, just to mention a few. But who can and do we want to trust with the integration of our data, with compiling a holistic picture of ourselves? Who can deal with all relevant regulations across different jurisdictions? “Big tech” of course offers an initial solution already with platforms such as Apple Health or Google Fit that in turn connect to these companies’ digital assistants, but many people will have justified privacy concerns. More advanced forms of privacy-preserving data sharing are still in their infancy (Becher et al. 2020) and suffer from technical complexity.

Third, monetization of health-related digital products is traditionally difficult (Bürk 2020). There are four basic routes to commercialization: One relies on end users paying the provider of the service directly, often through a subscription agreement. In today’s competitive market – there are over 45,000 health-related apps in Apple’s app store alone – finding enough paying customers is expensive. For example, Headspace, a relatively simple meditation app, has raised over $200 million in venture capital so far (Crunchbase 2020), and likely most of this money will go towards marketing. Another route relies on businesses paying for digital health products for their employees. This is often somewhat easier to achieve in the marketplace but comes with massive privacy concerns and the fear of stigmatization, since employees often suspect their employer of having at least partial access to their health and well-being data if they sign up for these services. The third route towards commercialization is reimbursement from health insurers. This is a notoriously slow and complicated method. Every health insurer in every country tends to have different standards, procedures and regulatory requirements for reimbursement of new health products. A pre-condition is typically some degree of clinical verification of the product, which is always expensive and in some use cases hard or nearly impossible to achieve. Finally, a fourth route are partnerships with pharma companies who are increasingly encouraged or even required by regulators to provide alternatives and complements to their pharmaceutical products, with digital health products being one of the options. This again is a time-consuming option with limited use cases.

These difficulties for even a limited scope of applications are an illustration of the need for a more comprehensive framework such as the one proposed by Österle. True improvements in quality of life supported by digital technology will only be possible if questions around privacy and ethics are resolved first in a holistic way. Furthermore, a truly interdisciplinary approach for the underlying science will be essential.

But equally important is the question of commercial feasibility and of who we will entrust with our most sensitive data in the context of a commercial service. The players who are currently in the best position to provide and commercialize integrated quality of life services – the big tech companies – are facing increasing backlash. Other types of established companies – health insurers and pharma companies in particular – seem to take a much more passive approach towards the topic. Plenty of startup companies are actively trying to solve a small part of the puzzle but lack the infrastructure and regulatory framework to integrate their services with others. There are no easy answers, but going beyond silos also in the commercial realm is a critical element that is still missing.

Using technology for the improvement of life, health and human well-being seems possibly to be the most important goal of digital innovation, not to mention probably one of the most interesting market opportunities in the history of technology. When it comes to digital technology applied to the quality of life, we are likely only at the very beginning of a long process. Österle’s integrated framework provides a useful and urgently needed way to think about these questions holistically which should help governments, regulators and commercial entities alike to contribute their part on this long journey.

## Life Engineering – Ethics or Quality of Life?

Hubert Österle

For decades, machine intelligence has changed companies and the economy. Now it is changing our lives. This generates fear and hope, while leading to a flood of discussions about ethics and quality of life (happiness and unhappiness) in all media and across all channels. What is the goal and how can we align development with it?

### Abundance and Fear Determine the Discussion

**Fig. 1:**
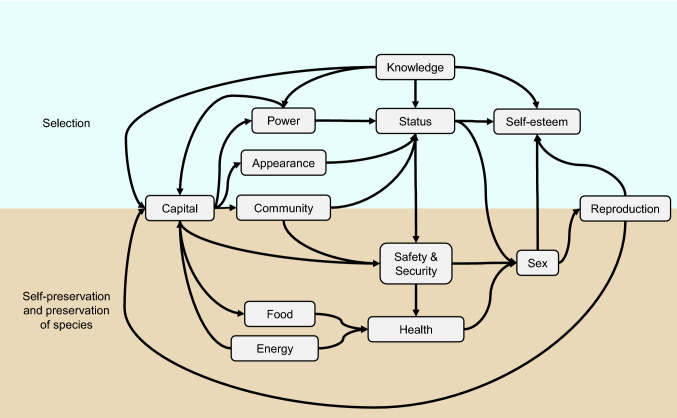
Network of needs

For highly developed societies at least, technology and capitalism have brought enormous material prosperity and satisfied needs such as food, security, and health, i.e. the needs of self-preservation and preservation of the species. But the affluent society can do more than satisfy basic needs. It gives the individual the opportunity to invest more energy in differentiation, as everyone tries to pass on their genes and to attract the most attractive reproductive partner. The needs of selection (see upper half in Fig. 1) come to the fore (Österle 2020c, p. 50 ff.) and drive human beings onto a treadmill in which, consciously or unconsciously, everyone is constantly working on their status, whether through clothing, offices in a club, knowledge, skills in music, youthful fitness, or simply through capital. An almost explosive growth in the literature on happiness research and ethics as well as an accompanying offer of lifestyle services such as happiness training, yoga, and wellness is aimed at helping us to gain as many positive feelings as possible from the satisfaction of all needs and to avoid negative feelings.

At the same time, there is a growing fear of what is to come. Dystopias such as surveillance capitalism, the totalitarian surveillance state, the loss of humanity and traditional values, or the excessive demands placed on the individual distract from the urgent task of shaping the coming change.

### Development Requires Ethical Guidelines

Phrases such as "for the benefit of humanity" have become a common element of corporate mission statements. But who actually believes in such statements? What has ethics, especially business ethics, as formulated by Heinrich Weber 100 years ago, actually achieved? It is certainly helpful to ask what kind of interests guide ethics.

### Companies and Business Leaders Want to Satisfy their Stakeholders

At the American Business Round Table (Business Roundtable 2019), nearly 200 CEOs of leading US companies signed a "fundamental commitment to all of our stakeholders". Many media articles have described it as an attempt to sugarcoat the social ills of digitalization through simple declarations of intent. Interestingly, the statement of these business representatives does not even mention the much more concrete international standard ISO 26,000 on Corporate Social Responsibility, which was adopted ten years ago. Digitalization requires many corporate leaders to demonstrate, among other things, responsible handling of personal data. Individual management consultants have reacted to this with offers for data ethics, aimed primarily at maintaining company ratings.

### Investors are Looking for Returns through Sustainability

Investors seek additional financial performance through investments that meet environmental and social criteria as well as the requirements of good governance (ESG – environment, social, and governance) (Schweitzer 2019). They try to identify the opportunities and risks of their investments at an early stage based on these criteria and thus increase the profitability of their investments. Rating agencies such as MSCI (MSCI 2020) and Inrate (Inrate 2020) evaluate listed companies according to ESG criteria for investors. In accordance with the recommendations of the OECD (OECD 2017), politicians use the weight of the financial markets to achieve sustainable development.

### Do-gooders Drive the Ethics Discussion

Avoiding the dangers of digitalization and seizing the opportunities for the benefit of human beings is a task for all citizens. Everyone must consider how they use digital services and what they expect from companies and politicians, for example, what personal data they give to Facebook, and where politicians should protect them from abuse. The danger arises when the discussion is dominated by do-gooders, who often argue purely emotionally, usually take only a very narrow partial view, and use vocal debate to compensate for their lack of knowledge and thus influence politics. Typical “enemies” are the greed of shareholders, totalitarian manipulation in China, the taxation of foreign corporations, and the “zombification” of mobile phone users. Do-gooders altruistically stand up for the good of the community but demand sacrifices mostly from others. In many cases, their commitment is a search for recognition of their efforts and a striving for self-esteem, which is often described as a “meaningful life” or similar phrases.

### Politics Follows the Need for Ethical Rules

Politicians need votes or the trust of their constituents. So they pick up on the popular mood and translate it into pithy catchphrases. A good example is the European Union’s announcement of the digital future of Europe (EC 2020c) with populist values such as fairness, competitiveness, openness, democracy, and sustainability. In addition to emphasizing fashionable topics such as artificial intelligence (AI), the paper focuses on the regulation of digitalization, while it hardly presents any concepts on how Europe should keep pace with the USA and China. The focus is on restricting entrepreneurial activity, not on exploiting potentials such as the Internet of Things (5G, sensor and actuator technology). The addressed citizens do not know these technologies or know them too little and they have neither the time, nor the motivation and the prerequisites to understand these technologies and their consequences. It is therefore much easier to evoke the previously mentioned enemy images than to arouse enthusiasm for misunderstood technologies.

This is also confirmed by the current discussion on the use of mobile phone users’ location data to curb the spread of Covid-19. The data that has long been used, for example, for planning public transport, is virtually negligible compared to the use of voluntarily submitted data on Google, Apple, or Facebook. Even classic personal data such as the traffic offenders' register in Flensburg, credit scorings, and customer data in the retail sector allow for far more dangerous misuse. Ethical values cultivated by do-gooders and attention-grabbing media make a serious discussion impossible on how the rapidly growing collections of personal and factual data could help to make human coexistence healthier, less conflictual, and more enjoyable (Österle 2020b) rather than concentrating on tightening criminal law.

### Ethics Wants Quality of Life for All

Ethics is looking for rules that should allow the highest possible quality of life for everyone. If we accept that digitalization cannot be stopped and that it will bring about massive socio-cultural change, we need mechanisms, now more than ever, to guide this change for the benefit of humankind. But do ethics and the underlying interests provide the tools? Two essential prerequisites are missing: First, ethics does not determine what quality of life actually constitutes. Second, there is a lack of procedures for objectively measuring quality of life.

A discipline called Life Engineering should start right there. It should develop a robust quality of life model based on the findings of psychology, neuroscience, consumer research, and other disciplines, and validate this model using the increasingly detailed and automatically collected personal and factual data. The network of needs can be a starting point if each of the needs, such as health, is broken down into its components, such as age, pain, weight, strength, and sleep quality, and the causal relationships are statistically recorded.

Once the factors of quality of life are better understood, it will be possible to better assess the opportunities and risks of digital services. The sensors of a smartwatch can measure possible influencing factors on health so that individualized correlations between physical activity and sleep behavior or heart rhythm disturbances can be recognized and the wearers of smartwatches can thus increase their health and well-being by taking simple measures. Such concrete, statistically sound evaluations of digital services currently remain the exception. However, a quality of life model, even in such a rudimentary form as the network of needs outlined above, provides at least a framework for discussion in order to evaluate technical developments in terms of arguments, as shown by the example of Instagram (Österle 2020a).

Ethics is based on values such as dignity, respect, trust, friendship, responsibility, transparency, and freedom, without justifying these values. However, such values are only relevant to people if they meet their needs and thus trigger positive or negative feelings. It is an exciting exercise to establish the relationship of ethical values like trust with needs like security, power, or efficiency (avoidance of effort).

It very quickly becomes clear how far away we are from a quality of life model that combines behavior, perceptions, needs, feelings, and knowledge. However, looking at the tasks of ethics, it is hardly justifiable not to at least try what is feasible. Right now, we are leaving this development to the megaportals, who try to understand and model these connections, but these companies e.g., Google (i.a. Selfish Ledger) (Burns 2018), and their management are being measured by their economic success, not by human quality of life. It is therefore almost inevitable that they will have to persuade customers to make the decisions that generate the most revenue.

Never before in the history of humankind have we had such comprehensive and automatically recorded datasets that allow statements about behavior and quality of life. The internet and sensors are documenting our lives more and more seamlessly, as Melanie Swan discovered as early as 2012 under the banner of the ‘quantified self’ (Swan 2012). The tools of machine learning and modeling in neural networks offer us the chance to recognize patterns of quality of life and to make them effective in digital assistants of all kinds, from shopping to nutrition, for the benefit of human beings. Never before has such intensive support been provided for people by machines in all areas of life through digital services. Never before has it been possible to give people such well-founded and well-targeted advice, to guide them in a recognizable but subtle way. The thought of this frightens the do-gooders and excites joyful expectation among the utopians.

With the methods of data analytics, health insurance companies evaluate the personal and factual data of their policyholders in order to better calculate the individual risks. They adjust the individual premiums in line with the individual risks, and ultimately reduce the claim costs of the insurer. For some policyholders, this leads to savings, but for those who are disadvantaged in terms of health and therefore in most cases financially less well off at the same time, it means higher payments. The redistribution of risk in the sense of solidarity is lost.

If an insurance company succeeds in better understanding the influences on health and – what is even more difficult – in guiding the insured to health-promoting behavior through digital services, then this machine intelligence helps both the insured and the insurers.

### Ethics Needs Life Engineering

Development cannot be stopped, but its direction can be influenced. We need a discipline called Life Engineering which translates the humanities concepts of traditional ethics and philosophy into design-oriented proposals, i.e. which pragmatically shapes technical, economic, and social development.

Only those who drive and lead development can influence it. The aversion to technology, which can be felt in many ethical discussions, has exactly the opposite effect to what it aims to achieve. It is therefore extremely welcome that engineers and AI architects, for example in the IEEE (Institute of Electrical and Electronics Engineers), come together to formulate rules for machine intelligence (IEEE 2019). Even without an elaborated quality of life model, it is possible to avoid at least some clearly unwanted characteristics of digital services. This calls among other things for rules stipulating that people can access the data stored about them and approve their use, or that a machine decision must be justified. However, these rules come up against the limitation of human cognitive abilities, i.e. whether a layman can even understand these connections within a reasonable time.

Apart from these obvious rules which do not have to be derived from scientific studies, it would be helpful if ethics could be based on an operational quality of life model. It is positive that version 2 of the IEEE guidelines on Ethically Aligned Design, unlike the first version, attempts to do just that. It is based on approaches and metrics for well-being. Its recommendations concerning the different aspects of ethics for machine intelligence ultimately provide a comprehensive agenda for Life Engineering.

In order to ever be able to meet such requirements, a Life Engineering discipline needs the following, in addition to financial resources:

- Access to the digital personal and factual data,
- Exchange of knowledge about behavior patterns and their effects on quality of life
- Access to the development of machine intelligence
- Political incentives for positive developments and prohibitions of negative developments

Life Engineering offers the chance to transfer ethics from the stage of a religion to a stage of science, just as the Enlightenment did in the eighteenth century. This has brought about a human development that probably only few people today would like to reverse.

## Towards a Human Life Engineering?

Edy Portmann

According to Österle (2020c), the emerging discipline of “Life Engineering” aims to utilize the potential of information technology to improve human quality of life. As a scientist of business informatics, Österle regards this as a development of Business Engineering, but with a focus on humans rather than on companies. And on top of this, Business Engineering is more concerned with materialistic progress, while the approach of Life Engineering rather helps to shape a digital but also sustainable future of mankind.

A focus point of the Swiss Post co-financed Human-IST Institute at the University of Fribourg is postal innovation. The collaboration between Swiss Post and the Human-IST Institute centers on the digitalization of network industries, where increasingly questions regarding digital ethics for the public sector arise. As the digitalization wave increasingly impacts not only markets but democracies, the network industries are seeking to digitalize their infrastructures for the benefit of Switzerland. Building on methods of business informatics (i.e., design-science research in information systems; Winter et al. 2008), the institute supports the postal network industry along its path.

In view of the growing importance of how human needs are reflected in technology and services, the collaboration is dedicating its efforts to the research and development of human-centered interaction science and technology (Human-IST) by developing sustainable interfaces for citizens that are usable, meaningful, attractive, trustworthy as well as consistent with high ethical values. Designing such technology implies to a certain degree also to engineer human life. An important point seems thus the alignment of the quality of life with digital ethics, so that a sustainable network infrastructure can be implemented in Switzerland.

To this end, with a human-centered approach, a kind of public service consortium research is conducted on an integrative basis into concepts, implementations, and the evaluation of new paradigms of interaction science and technology. Thus, over the past few years, multinational technology companies have continuously extended their business models, which are based on the collection and use of large volumes of data. Using all data, the companies not only continuously develop their models (e.g., with the aid of machine learning and artificial intelligence), but have also learned about our habits, attitudes, and values.

In this context, Zuboff (2019) talks about surveillance capitalism; thanks to supposedly free offers, we seem to have lost the sovereignty over our data. The services of the biggest player among the multinational technology companies (i.e., Amazon, Apple, Facebook, Google and Microsoft) are often financed by third parties that we do not know; with this strategy, these third-party companies expect to gain advantage from the information they can extract from our data. Besides impacting our markets, that also affects our democracies because, as well as gaining in-depth knowledge about us from our data, these companies are increasingly trying to manipulate us.

In democracies, governments are formed by holding political elections. Since power is exercised by the people as the general public, the freedom of opinion and of the press is key to the political decision-making process. In the light of the scandal surrounding Cambridge Analytica, which collected data from millions of US voters for the purpose of manipulating voter behavior by means of microtargeting using personally tailored information, democracy is increasingly under threat (Portmann 2020). Cambridge Analytica, which was forced to file for insolvency proceedings after the scandal, was headquartered in New York City, where it collected and analyzed the data relating to potential voters on a grand scale. With a one-sided digitalization of marketplaces and democracy, citizens’ data increasingly degenerate into the product that technology companies manage in their server farms.

To avert the threat to democracies, we need to build bridges on the one hand between research and practice and, on the other, also between different research disciplines. A nation forged by the will of the people (i.e., as in a democratic system) can only evolve through a broad debate with academia on the digitalization of society, of the public sector, and of the economy. If we are to design our “complex future with machines” (Ito 2019), we thus have to focus on integrative methods. This means going beyond (academic) disciplines and including the economy, the public sector, and society as a whole. In order to build bridges, human-centric research must extend beyond university boundaries by involving non-academic partners in the process. And while interdisciplinary research synthesizes knowledge from different existing disciplines, such a transdisciplinary approach combines all disciplines from research and practice to form one coherent whole.

Looking at things holistically and taking into account the interdependencies between them would appear to be a promising way of addressing the real complex problems of society. According to Ropohl (2005), transdisciplinarity is subject to a different paradigm than disciplinarity: Its challenges are often synthetic rather than analytical, which is why it uses first and foremost synthetic instead of analytical methods. However, this could enhance Österle’s (2020c) life quality model with humanistic values. For this reason, the methods of system theory represent suitable approaches to overcoming complex problems relating to how we understand and shape world policies. In this theory, aspects and principles of systems are used to describe and explain phenomena with different levels of complexity. A system denotes a coherent conglomerate of interlinked and interdependent parts, which can be either natural (e.g., a society) or artificial (e.g., a digital democracy).

Many of today’s systems are not researched holistically and are frequently based on binary thinking schemes and methods. As aids in “reducing complexity” (Ito 2019), such binary methods often rely on probability theory – despite the fact that they tend to be neither appropriate for nor representative of how humans think (e.g., Hobbs 1985). On the contrary, the truth is that hard facts appear to biological brains as gradual, fuzzy, vague, and to a certain extent inaccurate. Today, however, binary logic is reflected in all digitalization efforts (e.g., in machine learning, where the rounding up or down of – gradual fuzzy and vague – facts is used and tends to preserve, spread and reinforce this behavior; examples of this include the operation of applications by swiping right or left and clicking or not clicking a like button).

This becomes problematic when emotions are categorized as either positive or negative. The problem is that such categorizations leave no room for how and why one thing was selected as opposed to another. Decisions are seldom made on the basis of an absolute certainty. Humans tend to decide for or against something if they are largely convinced of it – or not – on the basis of their perceptions and attitudes. Related to this is the concept of perceptual computing (e.g., Seising 2007), which offers the potential to connect humanistic values with Life Engineering (and all its sustainable and ethical efforts). Today’s smart systems and artificial intelligence, however, only detects that a choice has been made and continues to work with this absolute statement, mostly without considering intermediate values. Focusing on fuzzy vocabulary (i.e., words, perceptions, etc.), a soft Life Engineering could break this chasm.

System theory, which is often seen as a basis for business informatics (Winter et al. 2008), relies on alternatives to binary thinking. According to Zadeh (in Seising 2007), however, complex systems cannot be properly analyzed with conventional methods, “because the description languages based on classical mathematics are not sufficiently expressive to serve as a means of characterization of input–output relations in an environment of imprecision, uncertainty and incompleteness of information” (p. 199). This means that traditional approaches can only model and analyze comparatively simple systems, while more complex systems (e.g., markets and democracies) frequently present an unresolvable task for conventional mathematical and analytical methods. The discipline of Life Engineering might face these limitations too. Hence, it could be upgraded proactively to a human-centered Life Engineering.

Zadeh states that, when developing intelligent systems, an important factor is the use of soft computing methods, in order to imitate the ability of the human brain, which works roughly rather than precisely, to effectively draw conclusions (Zadeh 1994). In combination with common methods of business informatics, these methods are therefore very appropriate when it comes to optimally addressing fuzziness. Soft methods enable attempts to understand (market and democratic) systems holistically (if only approximately; e.g., Portmann 2018). This means that experts can use such methods to design (soft) systems; they are able to bridge the gap between “soft” ethics and quality of life by making words and perceptions measurable and thus computable (e.g., Seising 2007). On this basis, a human Life Engineering endeavors to adopt a holistic system view in order to better capture the full spectrum of human lives.

As a transdisciplinary approach wants to achieve a synthesis of heterogeneous knowledge, it often has to grapple with language difficulties. For this reason, usually a core vocabulary (cf. computing with words and perceptions; Seising 2007) has to be developed first, which provides appropriate means of expression for knowledge syntheses. According to Ropohl (2005), system theory is suitable for this purpose: Soft computing therefore plays an important role in modeling the fuzzy core vocabulary of democracies by means of system theory. In doing so, it overcomes the frequently encountered binary logic using fuzzy logic (Portmann 2020).

By surrendering our personal data, we are potentially also giving away our democratic vote. Cambridge Analytica would not have been able to misuse data if citizens had been the owners of their data (Portmann 2020). But how can we protect citizens against enterprises of this kind? In human Life Engineering, the transformation of today’s systems into future ones, which digitalization inevitably entails, could be accomplished by means of alternative basic service mandates along with their respective digital trust models (e.g., for network industries like Swiss Post, which is owned by the confederation and where – thanks to our democracy – we are able to exert influence in the form of proposals). Instead of feeding the data models of multinational technology companies, we would then be taking over control of our data, which would enable us to safeguard our democratic values.

In their blueprint for a new digital society, Lanier and Weyl (2018) write that we should all own the copyright to our personal data, which in fact only exists because we do. In order to implement this, intermediaries should be created who attend to our data affairs on our behalf and whom we can trust. They refer to these as “mediators of individual data” (Lanier and Weyl 2018, p. 5). A digital network industry could assume a mediator role of this kind. In pursuing a new digital service mandate, it could help citizens to win back control over their digital self. In a data marketplace, the citizens would receive money – or other benefits – if their data was used for customer relationship management, marketing, or market research. Postal data mediators would make it possible to receive the appropriate return from data that the technology companies use for machine learning in a regulated and mediated marketplace of supply and demand. With a transdisciplinary approach, on behalf of Swiss Post, the Human-IST Institute explores such proposals in a practical way (i.e., by attempting to build smart interfaces that reflect our different languages, mindsets, values, cultures, qualities, and behavior to optimize our – collective – quality of life).

In his conclusion about democracies in the age of the Internet, Portmann (2020) wrote that “we are responsible for our democracy” (p. 4). In order to live up to that responsibility and therefore resist a factitious reduction of what it is to be human and consequently a reduction of our social systems (e.g., marketplaces and democracies), it is in our interests to explore soft computing methods that benefit us as citizens (cf. Portmann 2018). And what better way of addressing the digital metamorphosis of our public sector and the associated network infrastructure is there than than letting concepts inspired by human Life Engineering take the lead?

## The Value-based and Ethical Approach to Empower the Company and People

Sarah Spiekermann

Today’s life is increasingly penetrated by a digital fabric: how we socialize, meet, move, produce, think, speak – every activity in life seems to be interwoven with it. This digitization of life has consequences for the quality of our individual and social lives: for our mental and physical health, our identity formation, our intelligence as well as our future resilience at the personal, organizational and societal level. As digitization evolves with human aspirations that may be more or less wise, humans evolve as a consequence. For this reason, to engineer machines means to a certain extent to engineer life. If we get it wrong, we degrade and harm humanity, as even some Silicon Valley pioneers now realize (CHT 2020).

At the moment we are unfortunately getting it wrong, because the IT industry has been ignoring digitization’s fundamental impact on life, believing that it is somehow neutral. Notwithstanding the slow recognition that systems need to be usable, the embracing of truly positive human and social values––such as transparency, fairness, community, dignity or human autonomy––has remained more of an academic exercise than a matter of priority for corporate practice. It is true of course that security and privacy have recently fought their way onto corporate IT roadmaps. But this is probably not because corporations care so much for the after-effects of their systems, but because Sarbanes Oxley and a flood of security and privacy breaches have forced them to become more compliant with existing laws and international agreements.

That said, the reluctance towards ethics in IT design is dissolving on some fronts. With AI reaching Gartner’s hype cycle, a serious debate has been kindled around the values AI should respect (Jobin et al. 2019). No matter how much one believes in the myth of IT bringing the salvation of progress through its mere existence, nobody wants to buy dark science fiction stuff (except the military). As a result, a glimmer of hope is appearing on the horizon that ethics and values might finally establish themselves more firmly on the IT industry’s agenda. Long-existing branches of academic research, such as Value Sensitive Design, are suddenly being discovered (Friedman and Hendry 2019). The reductionist monetary meaning of the term “value” in twentieth century economics is being challenged. And in its place the original significance of “value” is restored, which denotes that a value bearer has a degree of worthiness, goodness or importance, so that it can be treasured in its own right. In this line of thinking, “Value-based Engineering” has emerged as a vision for a new era in engineering: an era that essentially strives to build systems and software such that they bear true progress for the lives of human beings, for organizations and society beyond profit (Spiekermann and Winkler 2020). The goal is that systems are worthy of being created not only because they generate profit or are somehow *useful* (as the "Technology-Acceptance-Model" has been emphasizing to utter excess), but because they contribute to a good, true, beautiful, peaceful and worthy life in which human beings can progress as individuals, unfolding their natural potentials instead of stifling them.

To live up to this ambition, Value-based Engineering fully “bases” the IT innovation practice on values and ensures that the resulting systems’ configurations are “based” on them. This “basing” of one’s system design effort on values is a very strong claim and goes much further than just saying that a system is “sensitive” to values. It requires Value-based Engineering to be more than a philosophy of design or a gentle stakeholder practice. Instead it is a rigorous step-by-step method for companies and public institutions to follow when they innovate: a guidance on how to go from an initial product idea to concrete specifications and deployment. It is a controlled and standardized path that responsible innovators can follow to systematically identify and strengthen the value proposition of their systems-of-interest (SOI) while ensuring that they do not step on stakeholder toes by breaching value expectations, laws or human rights.

When Value-based Engineering was first conceived with this vision (Spiekermann 2016) it benefited from its roots in German engineering culture, more specifically the Business Informatics discipline, which is respected for its long tradition in system modeling and system development in cooperation with industry. It became the starting point for IEEE’s 7000 Model Process for Addressing Ethical Concerns during System Design (IEEE, expected for 2021) and in many respects resembles this system engineering standard in the making (Spiekermann and Winkler 2020). However, knowing engineering methods and practical IT dynamics is not enough when it comes to “Life Engineering,” which should be a deeply ethical exercise. Humanity has over 4,000 years of records on ethical thinking and guidance on how to foster well-being and human flourishing; guidance, though, that differs widely across cultures. So any ethical or value-related engineering method should scale to the varying preferences of stakeholders using a system across the globe. It should respect and live up to this life diversity, and be ready to configure systems’ modes of operation with respect to target markets’ specific value preferences. Thinking this culture-specific system beauty to its logical conclusion implies that Value-based Engineering might move us from a quite homogeneous system landscape across the globe today to more heterogeneous system designs in the future. Also, the simplistic effort to work with preset lists of global value-principles is left behind. What is true, good and beautiful differs for every SOI, company and region of the world (except of course for some hygiene factors of responsible system design, such as reliability, privacy, security or transparency).

**Fig. 2:**
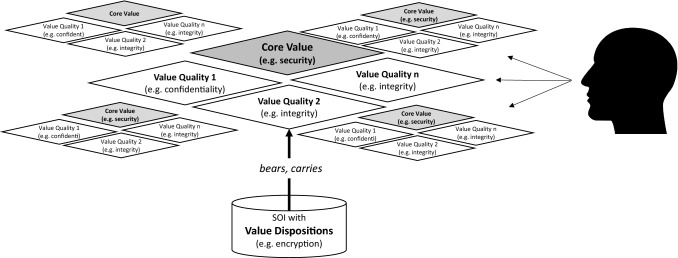
Value Ontology and Terms used in Value-based Engineering

To explicitly respect the diversity of value configurations in different contexts, Value-based Engineering is grounded in “Material Ethics of Value”, a stream of philosophy that is uniquely able to account for the phenomena an SOI incurs in its long-term real-life usage contexts (Scheler 1973; Hartmann 1932; Kelly 2011). Despite many contemporary efforts to study value dynamics, this twentieth century stream of philosophy seems to be not only the most elaborate one in existence to date, but one that resonates with timely advances in other “life-disciplines,” such as neuroscience/psychiatry (Fuchs 2017) and sociology (Rosa 2019). In line with the Material Ethics of Value, IT systems do not “have” values, and it will not be possible to build values “into” them. Instead engineers strive to build *value dispositions* into systems, so that in a subsequent second step *value qualities* can unfold in the eye of beholders (stakeholders). An example to clarify this ontologically important finesse is the value of security: An engineer will not build security “into” a system, but instead will create one or more value dispositions, such as the encryption of data. This encryption then *bears* the value quality of confidentiality. A human being – for example, a security expert – can appreciate this value quality. He or she might even *resonate* with a number of other positive value qualities, such as the integrity of the data and availability of the system, which exist due to other value dispositions built into it. Such a multitude of extrinsic value qualities appreciated by humans constitutes the higher intrinsic *core value* of security borne by the system. Figure 2 summarizes this ontological and terminological core of Value-based Engineering.

While proper terminology with philosophical grounding is an important prerequisite for any replicable ethical engineering method, it is not enough. Value-based Engineering is required to also offer a trustworthy way to overcome many additional challenges recognized by experts, two of which should be mentioned here: the first is to identify the right initial value priorities for an SOI; the second is to ensure that these value priorities are then traceably respected in the SOI design and deployment.

The first challenge, to determine what is right or wrong in a desirable future, is not done out of the blue, but is supported by the heterogeneous richness ethical theories have to offer. Note that in choosing these ethical theories, Value-based Engineering goes beyond the utilitarian tradition originating in Anglo-Saxon culture. Instead it embraces the classical virtue ethical forms of thinking as described by Aristotle (Aristotle 2000). And it also uses the Kantian deontological ethics to reflect about behavioral duties in order to identify and determine value priorities for system design. All this is done by including stakeholders from SOI target markets in a dialog that should be led by discourse ethical principles in order to openly reflect on cultural traditions that might help to anticipate a system’s value consequences not grasped by the Western-ethical canon. Taken together, four questions are asked for value elicitation:

1. What are the positive and negative life consequences one envisions from the SOI’s use for direct and indirect stakeholders? (Utilitarianism)
2. What are the negative implications of the SOI for the long-term character and/or personality of users – that is, which virtues or vices could result from widespread use? (Virtue Ethics)
3. Which of the identified values and virtues would you consider as so important (in terms of your personal maxims) that you would want their protection to be recognized as a universal law? (Duty Ethics)
4. Which forms of human conduct should be fostered by the SOI or prohibited, against the background of the religious, spiritual or common traditions of a target market?

Once values are thus elicited, they are prioritized and it is taken into account how important they are for life, human well-being and health. One possibility is that they may negatively impact life, human well-being and health, or are recognized in international human rights agreements and target market legislation. In this case, they must be traceably respected in the SOI’s design with the help of risk assessment methodology. Risk assessment methodology systematically anticipates likely value threats, followed by the identification of appropriate controls to address them similar to standards in security (NIST 2013) and privacy (EC 2011). The other possibility is that prioritized values do not impact meaningfully on human lives, but are nevertheless important in terms of strengthening the corporate value proposition. In this case they are set as the engineering goals pursued by any development method a company might have, including iterative or agile forms of work on prototypes. Value qualities are effectively becoming the goal function of these design efforts.

No matter what approach is taken, all value handling is captured in a Value Register and accompanied by some form of risk-thinking. That is, the engineering team keeps in mind that they should not risk forgoing a positive value proposition they actually agreed to prioritize or to undermine a value they found important. Finally, Value-based Engineering recognizes that value work never ends, as systems progress and evolve over time. Once a SOI is deployed into the real life of stakeholders, the values unfolding in reality are monitored and narratives are collected on what the true system impact is. Iteratively and over time, the SOI is then continuously improved to ensure it is and stays a good member of society.
